# Co-inoculation of broilers by *Campylobacter* and *Salmonella*: effect on colonization, cecal microbiota, and serum metabolome

**DOI:** 10.1128/spectrum.01102-25

**Published:** 2026-02-06

**Authors:** Muriel Guyard-Nicodème, Cyrielle Payen, Guillaume Larivière-Gauthier, Sophie Mompelat, Ségolène Quesne, Nagham Anis, Laetitia Bonifait, Laurent Guillier, Alassane Keita, Stéphanie Bougeard, Philippe Fravalo, Marianne Chemaly

**Affiliations:** 1 Hygiene and Quality of Poultry and Pork Products Unit, French Agency for Food, Environmental and Occupational Health and Safety (ANSES) 55036, Ploufragan, France; 2 USC Metabiot, Ploufragan, France; 3 Conservatoire National des Arts et Métiers (CNAM) 27054, Ploufragan, France; 4 Laboratory of Fougères, French Agency for Food, Environmental and Occupational Health and Safety (ANSES) 153855 , Fougères, France; 5 Risk Assessment Department, French Agency for Food, Environmental and Occupational Health and Safety (ANSES) 55036, Maisons-Alfort, France; 6 Avian Breeding and Experimental Department, French Agency for Food, Environmental and Occupational Health and Safety (ANSES) 55036, Ploufragan, France; 7 Epidemiology Health and Welfare, French Agency for Food, Environmental and Occupational Health and Safety (ANSES) 55036, Ploufragan, France; Health Canada, Ottawa, Canada

**Keywords:** *Campylobacter*, *Salmonella*, microbiota, metabolome, bacterial interaction

## Abstract

Campylobacteriosis and salmonellosis are the leading bacterial zoonoses in Europe, with poultry meat being the primary source of human contamination. Although both *Campylobacter* and *Salmonella* bacteria can coexist asymptomatically in chickens, their reciprocal impact remains underexplored. An *in vitro* study showed that *Campylobacter jejuni* survival was positively affected by the presence of *Salmonella*, but no data are available on this interaction in the animal gut. In this study, an *in vivo* investigation was carried out to explore the dynamics between *Campylobacter* and *Salmonella* colonization in chickens. The results revealed that both *Salmonella* and *Campylobacter* maintained significantly higher levels of colonization in the ceca throughout the experiment when co-inoculated compared to when inoculated alone. Additionally, changes in the microbiota were associated with each pathogen inoculated alone, but the simultaneous presence of *Campylobacter* and *Salmonella* induced specific modulations that could possibly explain this phenomenon. Significant differences were found in the serum metabolome of the contaminated groups, and partial least squares discriminant analysis models enabled the discrimination of contaminated animals from controls using these metabolic signals. Furthermore, possible links between variations in the microbiota and variations in the metabolome were identified.

**IMPORTANCE:**

This study demonstrates a synergistic effect between *Salmonella* and *Campylobacter jejuni* in the gut during co-infection in chickens, leading to an increased presence of both pathogens, as well as unique microbiota and metabolome changes. These findings underscore the importance of considering co-infection in poultry control measures and highlight the complex interplay between pathogens, microbiota, and metabolism.

## INTRODUCTION

With more than 140,000 and 70,000 human cases confirmed in Europe in 2023 for campylobacteriosis and salmonellosis, respectively, these two agents represent the main zoonoses of bacterial origin in the region (1). *Campylobacter jejuni* is responsible for more than 88% of human infections, and *Salmonella* Typhimurium is the second major serotype responsible for human infections (1). Poultry meat is a significant source of human contamination with these bacteria, and poultry often carry these pathogens asymptomatically in their gastrointestinal tract (1–3). While both *Campylobacter* and *Salmonella* can coexist in poultry intestines, no studies have explored how one pathogen might affect colonization by the other (4). According to the European Food Safety Authority, joint research and field investigations on these two bacteria are essential to better understand their dynamics and to develop more effective control strategies under field conditions (5). Moreover, a recent study suggested potential interactions between *Salmonella* Typhimurium and *Campylobacter jejuni*, noting that while *S*. Typhimurium did not influence the counts of *C. jejuni*, the survival of *C. jejuni* was enhanced by the presence of *S*. Typhimurium in aerobic growth conditions (6). However, it would therefore be interesting to determine whether this type of impact of the presence of *S*. Typhimurium could arise during the colonization of *C. jejuni* in the gut environment *in vivo*. Importantly, *in vivo*, the bacteria are subjected to complex ecological environments that must be taken into account in the interactions between these two pathogens. One of the factors that could affect this interaction is the presence of the gut microbiota. These two bacteria have been reported as being individually able to induce modulations in the composition of the intestinal microbiota of poultry. This mainly resulted in a change in β-diversity (7–10). *Salmonella* Typhimurium was, for example, able to increase the abundance of *Proteobacteria* in the microbial consortium (8, 11) and to variably modulate the abundance of lactic acid or short-chain fatty acid (SCFA)-producing bacteria (7, 11, 12). *Campylobacter jejuni* was also able to increase the abundance of *Proteobacteria* (13, 14) and decrease the abundance of lactic acid-producing bacteria such as *Lactobacillus* and *Bifidobacterium,* as also observed with *Salmonella* (13, 15, 16). However, to our knowledge, no study has investigated the effect of *Campylobacter* and *Salmonella* co-inoculation on the composition of the gut microbiota of poultry.

The metabolome is the complete collection of small molecules (<1 kDa) produced during metabolism (17). Serum metabolome can be altered by bacterial infection in animals or humans through the disruption of physiological processes and activation of the immune system (18, 19). In addition to host-induced metabolic changes, one of the potential sources of metabolome variation is the bacteria composing the digestive tract microbiota. These bacteria are responsible for the production of multiple molecules that are part of the normal metabolome or can have an impact on their production by the host itself (20–22). Some of these metabolites produced in the gut can be absorbed into the bloodstream. As a result, variations of the metabolites produced in the gut can also affect the metabolome of samples from distant sites of the body (liver, kidney, serum) (23, 24). Hence, changes in gut microbiota following an enteric infection have been shown to cause variation in the global host metabolome. For example, the impact of inoculation of chickens by *Salmonella* on the cecal and serum metabolome has already been shown to be linked to variations in the microbiota (18, 25). However, while the impact of an infection by *S*. Typhimurium on the serum metabolome of neonatal chicks has been described, no information on its impact on long-term asymptomatic contamination is available (18). Furthermore, to our knowledge, no information is available on the effect of *Campylobacter* individually or in co-inoculation with *Salmonella* on the serum metabolome of chickens, and the link between variations in the microbiota and metabolome in these cases has not been studied.

This study aimed to investigate the impact of *Campylobacter* on the colonization of *Salmonella* and *vice versa,* in an *in vivo* model. In addition, the research sought to investigate the effect of *Campylobacter* and *Salmonella,* both individually and in combination, on the composition of the cecal microbiota in poultry and on the serum metabolic signature of the animals over time. Finally, the study aimed to establish correlations between variations in the composition of the intestinal microbiota in each condition and corresponding changes in the serum metabolic signature.

## MATERIALS AND METHODS

### *Campylobacter* and *Salmonella* strains and growth conditions

Cultures of *C. jejuni* strain C97Anses640 were carried out as follows. The bacteria, stored at −80°C, were thawed and plated on selective modified charcoal cefoperazone deoxycholate agar (mCCDA; Thermo Fisher Diagnostics SAS, Dardilly, France) and incubated for 48 h under microaerobic conditions (85% N_2_, 10% CO_2_, and 5% O_2_) at 41.5°C. Then, one colony from this plate was spread on another mCCDA and incubated for 48 h under the same conditions. One colony from this last plate was then inoculated into Brucella broth (BB; Becton Dickinson, Le Pont-de-Claix, France) for 24 h under the same conditions. The concentration of the bacterial suspension was adjusted to approximately 10^5^ CFU/mL in Tryptone salt broth (TS; bioMérieux, Marcy-l’Etoile, France) and confirmed by enumeration on mCCDA.

Cultures of *S*. Typhimurium HQPAP97FF/022 CNEVA, a rifampicin-resistant strain stored at −80°C, were thawed and plated on xylose lysine deoxycholate (XLD; Thermo Fisher Diagnostics SAS, Dardilly, France) supplemented with rifampicin (XLD-rif) at a concentration of 100  mg/L rifampicin (Sigma Aldrich, Saint-Quentin-Fallavier, France) and incubated for 24 h at 37°C. Then, one colony from this plate was spread on a plate count agar (PCA; Biokar Diagnostics, Allonne, France) and incubated for 24 h at 37°C. One colony from this plate was then inoculated into brain heart infusion (BHI; Biokar Diagnostics, Allonne, France) and incubated for 12 h at 37°C. The concentration of the bacterial suspension was confirmed by enumeration on XLD-rif. The BHI containing bacteria was then adjusted to approximately 10^5^ CFU/mL in TS.

### Experimental design and sampling

The experiment was carried out in accordance with institutional guidelines on the care and use of animals for research at the French Agency for Food, Environmental and Occupational Health and Safety (ANSES), Ploufragan Laboratory (France), which has an agreement for animal experimentation (No. E-22-745-1).

**Fig 1:**
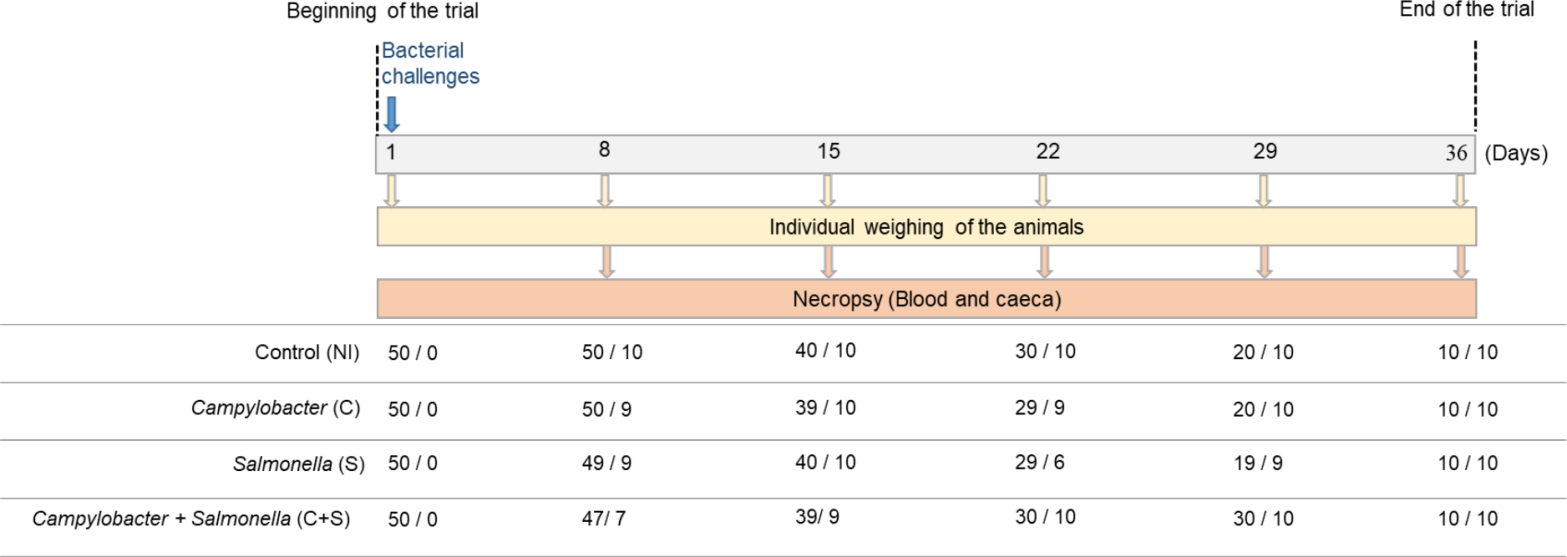
Diagram presenting the main steps of the experimental design for the *in vivo* trial. The number of broilers used for individual weighing (chickens remaining in the trial) and for necropsy is presented (individual weighing/necropsy) for each sampling date. The number of days in the trial corresponds to the age of the chicks.

The study was carried out using 200 × 1-day-old conventional broiler chicks (Ross 308, without gender selection) purchased from a local hatchery. Five additional chicks and transport crates were sampled to confirm the *Salmonella* spp.-free status and thermotolerant *Campylobacter* status before the beginning of the experiment. The 200 chicks were randomly allocated into four groups of 50 chicks, with each group being placed in a separate room: Non-inoculated control group (NI), *Campylobacter*-challenged group (C), *Salmonella*-challenged group (S), and *Campylobacter + Salmonella*-challenged group (C + S). Birds were housed in 3.4 m^2^ floor pens (1.85 × 1.85 m) and kept under a 12:12 (L:D) lighting cycle. All animals were fed *ad libitum* with a starter grower diet from days 1 to 19, followed by a grower-finisher diet until day 36. Broilers had free access to drinking water. On the same day (D1), chicks from the NI, C, S, and C + S groups were orally administered 100 µL of TS, 100 µL of a suspension containing 10^5^ CFU/mL of *C. jejuni* C97Anses640, 100 µL of a suspension containing 10^5^ CFU/mL of *S*. Typhimurium, or 100 µL of a suspension containing 10^5^ CFU/mL of *C. jejuni* strain C97Anses640 and 10^5^ CFU/mL of *S*. Typhimurium, respectively. On D8, D15, D22, D29, and D36, all the animals were weighed, and then 7–10 birds were randomly selected from each group and euthanized for sampling (Fig. 1). Broilers were euthanized by electronarcosis anesthesia, followed by bleeding. During the bleeding, a maximum of 15 mL of blood was collected to study the metabolome; then, the cecum of each animal was recovered aseptically to study the microbiota, and for *Campylobacter* and *Salmonella* detection and enumeration.

### Thermotolerant *Campylobacter* spp. detection and enumeration

Thermotolerant *Campylobacter* spp. were detected using cecal samples from the four animal groups by direct plating on modified charcoal cefoperazone deoxycholate agar (mCCDA, Thermo Fisher Diagnostics, Dardilly, France) and *Campylobacter* selective agar (CASA; bioMérieux, Marcy-l’Etoile, France). The presence of typical *Campylobacter* colonies was determined after incubation under microaerobic conditions (85% N_2_, 10% CO_2_, and 5% O_2_) at 41.5°C for 48 h. *Campylobacter* spp. were enumerated only from the cecal samples of the C and C + S groups after serial dilution (1:10 vol/vol) in buffered peptone water (BPW; bioMérieux, Marcy-l’Etoile, France) and plating on mCCDA and CASA. The plates were incubated under microaerobic conditions at 41.5°C for 48 h. Then, typical *Campylobacter* colonies were counted and converted to log10 CFU/g of cecum content. Counts performed on CASA and mCCDA gave equivalent concentrations of *Campylobacter*. For each sample, the average of the concentrations obtained for mCCDA and CASA was calculated. The enumeration threshold of *Campylobacter* was 2 log10 CFU/g.

### *Salmonella* detection and enumeration

*Salmonella* spp. were detected using cecal samples from the four animal groups diluted 1:10 vol/vol in BPW. After incubation for 18 h at 37°C in aerobic conditions, this solution was used to inoculate selective enrichment broths: 1 mL on Muller-Kauffmann tetrathionate-novobiocin broth (MKTTn; Biokar Diagnostics, Allonne, France) and 100 µL on modified semisolid Rappaport-Vassiliadis agar (MSRV; Biokar Diagnostics, Allonne, France). MKTTn broth and MSRV agar were incubated at 41°C for 24 h. If no characteristic migration was observed on MSRV agar after 24 h of incubation, the MSRV agars were incubated for an additional 24 h. MKTTn broths were streaked on XLD and Rapid’Salmonella (R’S; Bio-Rad, Marnes-la-Coquette, France) and incubated for 24 h at 37°C. A sample was considered positive if typical *Salmonella* colonies were observed on XLD and R’S and if characteristic migration was observed on MSRV agar. *Salmonella* spp. were only enumerated from the cecal samples of the S and C + S groups after serial dilution (1:10 vol/vol) in BPW (bioMérieux, Marcy-l’Etoile, France) and spread on R’S supplemented with rifampicin (100 mg/L; Sigma Aldrich, Saint-Quentin-Fallavier, France). The plates were incubated at 37°C for 24 h in aerobic conditions, and typical *Salmonella* colonies were then counted and converted to log10 CFU/g of cecum content. The enumeration threshold for *Salmonella* was 2 log10 CFU/g.

### Microbiota study

For each cecal sample, 1 mL of 1:10 diluted cecum content in BPW was centrifuged at 10,000 × *g* for 10 min, and the supernatant was gently removed before storage of the samples at −80°C until use. Bacterial DNA was extracted as described by Gloanec et al. (26).

The V3-V4 hypervariable regions of the 16S rDNA gene were amplified using forward primer: 5′-TCGTCGGCAGCGTCAGATGTGTATAAGAGACAGCCTACGGGNGGCWGCAG-3′ and reverse primer: 5’- GTCTCGTGGGCTCGGAGATGTGTATAAGAGACAGGACTACHVGGGTATCTAATCC-3′. An Illumina MiSeq sequencer was used to perform 2 × 300 bp paired-end sequencing. An Illumina MiSeq reagent kit 600 v3 was used, according to the Illumina 16S metagenomic library preparation protocol (15044223 Rev B adapted) and the MiSeq system denature and dilute libraries guide (15039740-3 December 2017 adapted). All sequence processing was performed using FROGS (Version 4.0.1 +galaxy1) (27). Each sampling series was treated independently to avoid the loss of data that could be induced by the lack of richness and diversity in the first week. Briefly, paired-end reads were merged using VSEARCH. Sequences were filtered by their length (380–500 nucleotides), by removing those with ambiguous bases and those without a primer sequence at both the 3′- and 5′-ends (no mismatch allowed). Swarm was used to cluster amplicon sequence variants (ASVs). VSEARCH was used to remove chimeras. ASVs were excluded when they were not present in at least two samples and when representing less than 0.005% of the total number of sequences (28). Contaminating sequences were excluded using the phiX databank. The BLAST algorithm was used for taxonomic affiliation with the SILVA 16S database (Pintail80, version 138.1).

### Metabolome study

For each animal, serum was collected from coagulated blood samples by centrifugation at 3,500 × *g* for 10 min (4°C) and then stored at −80°C until use. Extraction of metabolites and data processing were performed as previously described in reference 29.

Metabolites were extracted by adding 100 µL of serum to 500 µL of a cold (−20°C) 50/50 volume-to-volume ratio methanol and acetonitrile solution (Thermo Fisher Scientific, Illkirch-Graffenstaden, France). The mixture was vortexed for 30 s and left to stand at −20°C for 30 min. The solution was then centrifuged at 15,000 × *g* for 10 min at 4°C. Afterward, the supernatant was collected and subjected to complete evaporation under nitrogen flow in a TurboVap evaporator (Biotage, Uppsala, Sweden) at 30°C. The extracts were redissolved in a solution of 100 µL 0.1% formic acid and methanol (98/2 volume-to-volume ratio) (Supelco, Saint-Quentin-Fallavier, France). Following this step, 50 µL of a solution containing nine isotopically labeled internal standards was added to each sample. Prior to being transferred to liquid chromatography–mass spectrometry (LC-MS) conical vials, the samples were filtered using a 0.22 µm filter. In addition, quality control samples (QCs), which represent the entirety of metabolites present in the individual samples, were prepared. This was achieved by pooling 10 µL of each sample extract.

The metabolomic data acquisition was executed using high-performance liquid chromatography combined with high-resolution mass spectrometry (HPLC–HRMS), using an electrospray ionization source operating in positive ionization mode. The process of chromatographic separation was carried out via the ultra-high-performance liquid chromatograph (U-HPLC) Vanquish Flex (Thermo Fisher Scientific, Illkirch-Graffenstaden, France) using an Accucore Phenyl-Hexyl column (100 × 2.1 mm, 2.6 µm, Thermo Fisher Scientific, Illkirch-Graffenstaden, France) maintained at a flow rate of 300 µL/min and a consistent temperature of 30°C for a duration of 30 min. The mobile phase gradient fluctuated from 2% methanol (Optima, LC–MS grade, Thermo Fisher Scientific, Illkirch-Graffenstaden, France) in water with 0.1% formic acid (Supelco, Saint-Quentin-Fallavier, France) to a composition of 98% methanol. Mass spectrometry detection was conducted using a Q Exactive Plus Hybrid Quadrupole-Orbitrap mass spectrometer (Thermo Fisher Scientific, Illkirch-Graffenstaden, France). For each sample, a volume of 5 µL was injected in an arbitrary sequence, and for every set of five samples, one QC sample was injected to maintain a check on the variability of the analytical system. Two blank samples, incorporating all the extraction reagents but without the serum, were also injected at the beginning and end of the experiment. The spectra of all samples were captured in full scan mode.

Data preprocessing was conducted using the XCMS software package, version 3.12.0, executed on the workflow4metabolomics (W4M) galaxy platform (30). Chromatographic peak detection was carried out using the findChromPeaks function and the centWave method. The maximum allowed ppm m/z deviation in sequential scans was established at 15, with the peak width confined between a minimum of 10 and a maximum of 60 s. The smallest acceptable m/z difference for peaks sharing overlapping retention times was set to 0.015. All other settings were kept at their standard values. Peak grouping was performed using the peak density method under the groupChromPeaks function, with a bandwidth setting of 5, while all other parameters remained at their default values. Variations in retention time were corrected using the PeakGroups method within the adjustRtime function, using the standard settings. A second round of peak grouping was carried out post-retention time correction, using the same set of parameters. Ultimately, any missing peaks were filled using the fillChromPeaks function.

Subsequently, the Collection of Algorithms for MEtabolite pRofile Annotation (CAMERA) package was employed for the annotation of isotope peaks, adducts, and fragments in peak lists. Signal drift was rectified using the Batch_correction function of the workflow4metabolomics pipeline, employing the all-pool method. In this approach, a regression model is fitted to the values of the pools (QC samples) and used to calibrate the intensity of the metabolic signals in the samples. Features that did not yield a signal intensity value (NA) for all samples were discarded. Also, those displaying a coefficient of variation exceeding 30% in the QC samples were omitted, as well as features that had an intensity in the QC samples less than triple the intensity in the blank samples. Metabolites correlation analysis was also carried out to identify correlated features that correspond to different ions of the same molecule. For these correlated features, only the features exhibiting the highest intensity were retained, while the others were removed.

To identify possible outliers, samples were compared using Hotelling’s t-squared distance. Samples for which the statistical tests showed a highly significant result (*P* < 0.001) were eliminated from the data set.

The values of intensity of the metabolic signals were log 2 transformed and normalized using the total intensity method of W4M for each sample. Finally, to reduce background noise caused by metabolic signals that did not vary significantly, a Kruskal–Wallis test was conducted on all the signals for each week post-inoculation separately, and only signals that had a significantly different intensity (*P* < 0.05) in at least one group were kept for further analysis.

### Statistical analysis

A *P*-value lower than 0.05 (*P* < 0.05) was considered statistically significant.

#### Body weight of animals

For each time point, comparisons between the groups were performed using an analysis of variance (ANOVA) test, followed by a Tukey test when the data met the criteria for parametric tests; otherwise, a Kruskal–Wallis test followed by Mann–Whitney tests was performed.

#### Bacterial enumeration

For bacteriological comparison of *Salmonella* and *Campylobacter* levels, Student’s or Mann–Whitney tests were carried out, depending on whether the distribution satisfied requirements for parametric tests.

#### Microbiota

The FROGS Phyloseq R package (available in the FROGS pipeline) was used to perform the statistical analyses of the microbiota for each week (31). The samples were rarefied to an even sequencing depth for α and β diversity analyses. Observed ASVs and Shannon indices obtained for samples from the four groups were compared using a one-way ANOVA test, after verifying that the distribution was normal, followed by a Bonferroni’s multiple comparison test. β diversity was analyzed using the Jaccard distance matrix and visualized with a principal coordinate analysis (PCoA). Permutational multivariate analysis of variance (PERMANOVA) using an ADONIS pairwise test was performed to test the influence of the bacterial challenge on chicken cecal microbiota.

Then, a linear discriminant analysis (LDA) effect size (LEfSe) method (32) was used to compare the C, S, and C + S groups to the NI group (for analyses, only significant taxonomic ranks with an LDA score over two were reported).

#### Metabolome

The impact of the various challenges on the global composition and intensity of the metabolic signals found in the samples was evaluated using multivariate analysis. The Euclidean distance between the samples of the different experimental groups for each week post-inoculation was calculated. These distances were visualized using PCoA, and the different groups were compared statistically using a PERMANOVA test. Euclidean distance calculation and PERMANOVA tests were conducted using the vegdist and adonis2 functions of the vegan R package.

The possibility of classifying the animals in their experimental groups using their global serum metabolome was also evaluated using partial least squares discriminant analysis (PLS-DA) models. The differences between the groups were considered significant when (i) the R^2^Y and Q^2^ values for the model were of similar amplitude and with values over 0.600 and (ii) the permutation tests conducted on each of these values were significant (*P* < 0.05). These analyses were conducted using the ropls R package: opls function with the orthoI (number of orthogonal components) option set to 0 for PLS-DA.

Signals that mainly influenced the discrimination of the different groups were identified using bootstrapped sparse PLS-DA models (sPLS-DA). Sparse models used LASSO penalization to reduce the number of variables used in the models, by selecting those that best discriminate between groups. For each comparison, the models were produced 500 times using, for each iteration, a new data set created by random sampling with replacement, while keeping the proportions of the compared groups. The variables that were selected for the creation of the sPLS-DA models more than 60% (300/500) of the time were considered determinant. These analyses were conducted with the mixOmics 6.19.1 package under R, using the perf function to select the optimal number of components, and the tune.spls function selects the optimal number of variables, and the spls function builds the final models. The bootstrapped data set was created using the sample() function, with the replace argument set to TRUE.

#### Correlations between microbiota and metabolome

Changes in cecal microbiota in the different groups that could explain variations in serum metabolic signals were explored. Variables that were used to build these correlation models were the metabolic signals that were considered important in the bootstrapped sPLS-DA models and taxa (at the genus level) whose relative abundance varied significantly between at least one group of inoculated and control animals in a week. A link between a genus and a metabolic signal variation was considered important when a positive association (higher than 0.45) or negative association (lower than −0.45) was obtained in the final model. These analyses were conducted with the mixOmics 6.23.4 package (33) under R, using the perf function to select the optimal number of components, and the tune.spls function selects the optimal number of variables, and the spls function builds the final models.

## RESULTS

### Effect of the different bacterial challenges on animal’s mean body weight

The mean animal body weight was not different between the groups at the beginning of the study. Weight in the group challenged with *Campylobacter* alone (C) was not significantly different (*P* < 0.05) from that in the non-inoculated control group (NI) throughout the study. However, 1 week after bacterial challenge (D8), the mean body weight in both the S and C + S groups (Table 1) was significantly lower than that in the other two groups. Additionally, from D15, the mean body weight in the S group was not different from that in the NI and C groups (Table 1). For the C + S group, the mean body weight remained lower than that in the other groups until the end of rearing, with significant differences especially at D15 and D29 (Table 1).

**TABLE 1:** Mean body weight in g (mean ± SD) of broilers from the different experimental groups at several time points during the trial^*a*^

Day	Experimental group			
	NI	C	S	C + S
D0	44 ± 3^a^ (*n* = 50)	43 ± 3^a^ (*n* = 50)	43 ± 3^a^ (*n* = 50)	44 ± 3^a^ (*n* = 50)
D8 (week 1)	220 ± 17^a^ (*n* = 50)	218 ± 22^a,b^ (*n* = 50)	206 ± 26^c^ (*n* = 49)	196 ± 25^c^ (*n* = 47)
D16 (week 2)	552 ± 46^a^ (*n* = 40)	566 ± 47 ^a,b^ (*n* = 39)	531 ± 66 ^a,b,c^ (*n* = 40)	502 ± 73^c^ (*n* = 39)
D22 (week 3)	1100 ± 99^a^ (*n* = 30)	1079 ± 106^a^ (*n* = 29)	1084 ± 169^a^ (*n* = 29)	1026 ± 107^a^ (*n* = 30)
D29 (week 4)	1852 ± 205^a,b,c^ (*n* = 20)	1818 ± 200^a,b,c^ (*n* = 20)	1915 ± 192^b^ (*n* = 19)	1741 ± 203^c^ (*n* = 19)
D36 (week 5)	2778 ± 497^a^ (*n* = 10)	2640 ± 321^a^ (*n* = 10)	2818 ± 300^a^ (*n* = 10)	2518 ± 398^a^ (*n* = 10)

^*a*^ The number of chickens (*n*) in each group is indicated in parentheses. For each time point, comparisons between the groups were performed using an ANOVA test followed by a Tukey test when the data met the criteria for parametric tests (D29, D36); otherwise, a Kruskal–Wallis test followed by Mann–Whitney tests was performed (D0, D8, D16, D22). Different superscript letters indicate a significant difference (*P *< 0.05) between the groups. NI: non-inoculated broilers (control group); C: broilers challenged with *Campylobacter*; S: broilers challenged with *Salmonella*; C + S: broilers challenged with *Campylobacter* + *Salmonella*.

### Colonization of broilers after a challenge with *Campylobacter* and *Salmonella* alone or in combination

**Fig 2:**
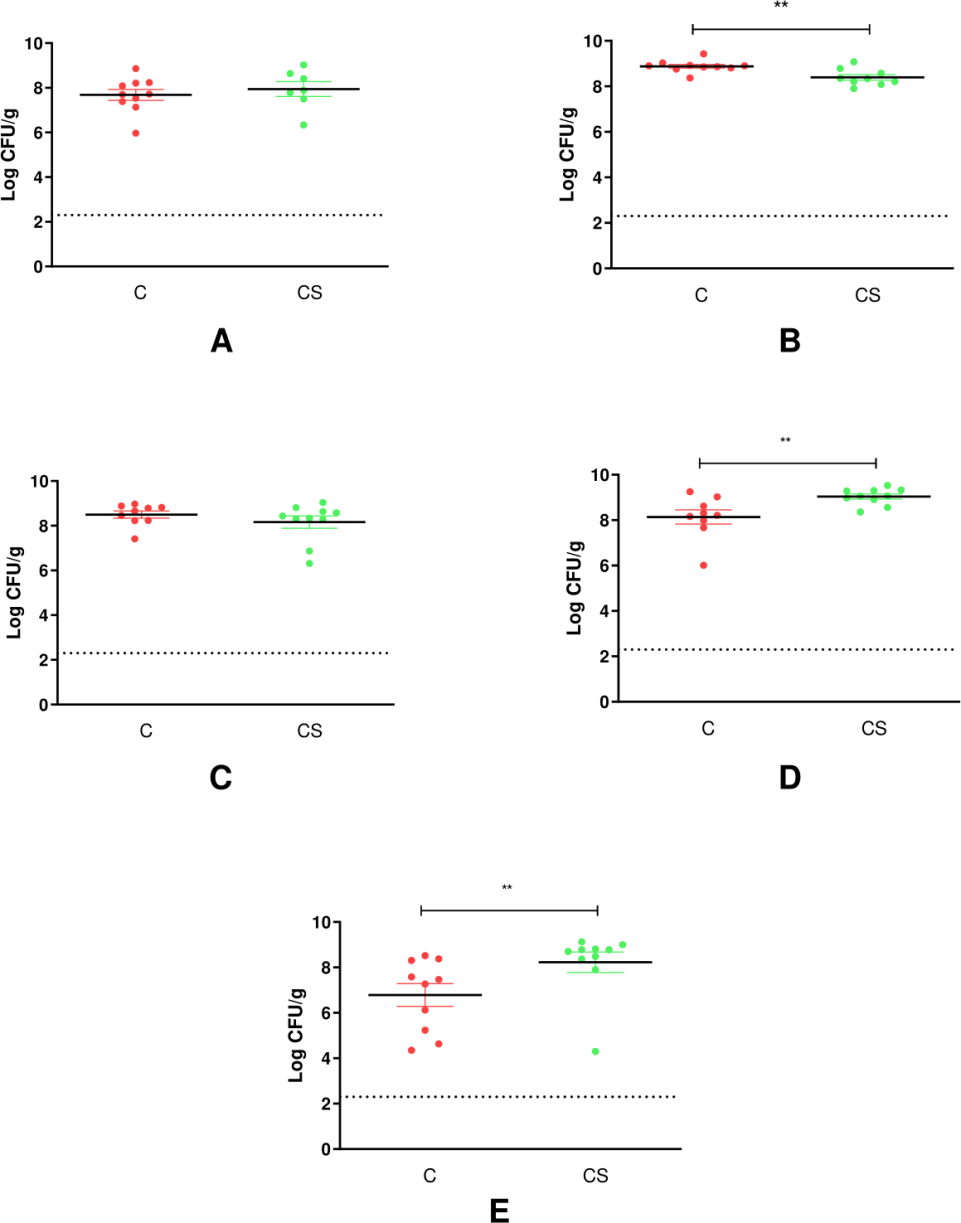
*Campylobacter* colonization of broiler cecum at (**A**) week 1, (**B**) week 2, (**C**) week 3, (**D**) week 4, or (**E**) week 5 post-inoculation with *Salmonella* alone (S) or *Campylobacter + Salmonella* (C + S). **P < 0.01; Student’s test was performed when the assumptions of normality and homogeneity of variances were met (weeks 1 and 2); otherwise, the Mann–Whitney test was performed (weeks 3, 4, and 5).

On D8, 1 week after bacterial challenge, the cecal enumeration of *Campylobacter* was equivalent between the C and C + S groups, at 7.7 ± 0.8 log_10_ CFU/g and 7.9 ± 0.9 log_10_ CFU/g (*P* = 0.53), respectively. However, at the end of the study (D36), the cecal level of *Campylobacter* was significantly higher for broilers from the C + S group compared to that in the C group (C: 6.8 ± 1.6 log_10_ CFU/g vs C + S: 8.2 ± 1.4 log_10_ CFU/g; *P* = 0.047) (Fig. 2).

**Fig 3:**
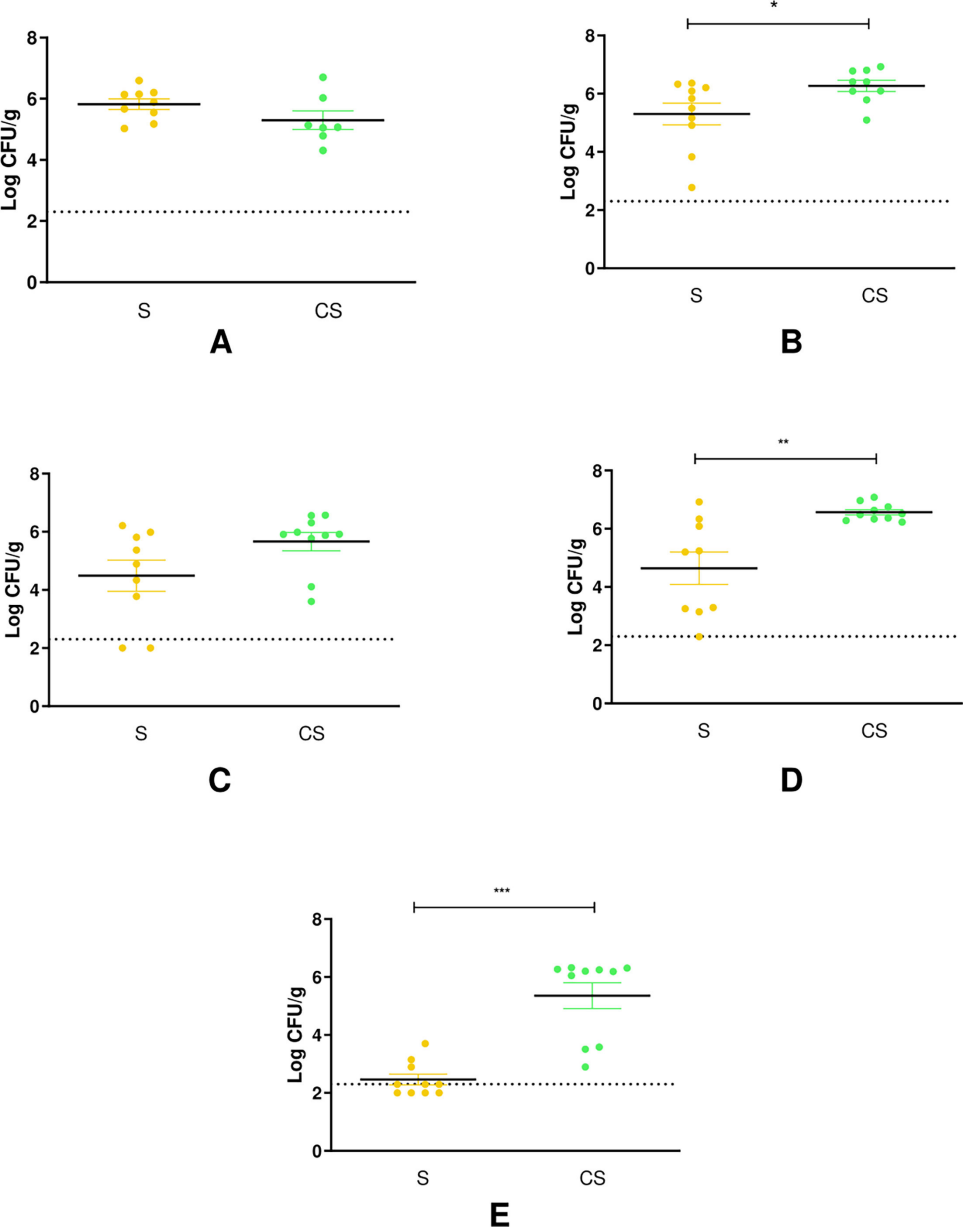
*Salmonella* colonization of broiler cecum at (**A**) week 1, (**B**) week 2, (**C**) week 3, (**D**) week 4, or (**E**) week 5 post-inoculation with *Salmonella* alone (S) or *Campylobacter + Salmonella* (C + S). *P < 0.05; **P < 0.01; ***P < 0.001; Student’s test was performed when the assumptions of normality and homogeneity of variances were met (weeks 1 and 2); otherwise, the Mann–Whitney test was performed (weeks 3, 4, and 5).

On D8, the cecal level of *Salmonella* was 5.8 ± 0.5 log_10_ CFU/g in the S group and decreased to 2.5 ± 0.6 log_10_ CFU/g at the end of the trial on D36. However, when *Salmonella* was co-inoculated with *Campylobacter* (C + S), the cecal level of *Salmonella* was maintained throughout the study. At D8 and D36, *Salmonella* enumeration was 5.3 ± 0.8 log_10_ CFU/g and 5.3 ± 1.4 log_10_ CFU/g, respectively, in this group. The cecal level of *Salmonella* was significantly higher in broilers from the C + S group compared to that in the S group at D15, DP29, and DP36 (*P* = 0.04, *P* = 0.0022, and *P* < 0.001, respectively) (Fig. 3).

These results indicate that animals challenged with both bacteria (C + S) had higher numbers of *Campylobacter* and *Salmonella* at the end of the trial compared to when they were challenged with only *Campylobacter* or *Salmonella*.

### Changes in cecal microbiota in response to bacterial challenges

The 16S metabarcoding sequencing generated between 1,664,605 and 4,196,650 paired-end reads, from week 1 to week 5. After processing, these sequences generated between 346 clusters as ASVs at week 1 and 732 at week 5. Four bacterial phyla were identified in the cecal microbiota throughout the study. *Firmicutes* and *Proteobacteria* were the major phyla. From week 1 to week 5, the relative abundance of *Firmicutes* increased from 70.6% to 83.6%, while the relative abundance of *Proteobacteria* decreased from 26.6% to 12.1%. The *Campylobacterota* phylum emerged from week 1, with relative abundance of 2.6% and 3.8% at week 5, respectively, and reached a maximum of 8.7% at week 2. However, this phylum was only detected in the groups with animals challenged with *Campylobacter* (C and C + S). *Actinobacteriota* was also identified, but the relative abundance of this phylum represented 0.1% at week 1 and 0.4 % at week 5 (Table S1). Considering all the groups, the four major bacterial genera identified from ceca at week 1 were *Ruminococcus torques group* (27.7%)*, Escherichia-Shigella* (23.0%), *Clostridium_sensu_stricto1* (11.1%), and *Erysipelatoclostridium* (10.8%) (Fig. S1). At week 2, *Ruminococcus torques group* (24.7%)*, Escherichia_Shigella* (17.4%), (8.7%), and *Erysipelatoclostridium* (4.3%) were the major bacterial genera identified (Fig. S1). At week 3, *Ruminococcus torques group* (13.7%)*, Escherichia-Shigella* (11.6%)*, Campylobacter* (6.2%), and *Eisenbergiella* (4.8%) were the major bacterial genera identified (Fig. S1). At week 4, *Escherichia-Shigella* (10.0%)*, Ruminococcus torques group* (8.7%)*, Campylobacter* (6.3%), and *Blautia* (5.3%) were the four major bacterial genera identified (Fig. S1). Finally, at week 5, *Escherichia-Shigella* (11.6%), *Faecalibacterium* (10.2%), *Blautia* (4.8%), and *Limosilactobacillus* (4.4%) were the four major bacterial genera identified (Fig. S1).

**Fig 4:**
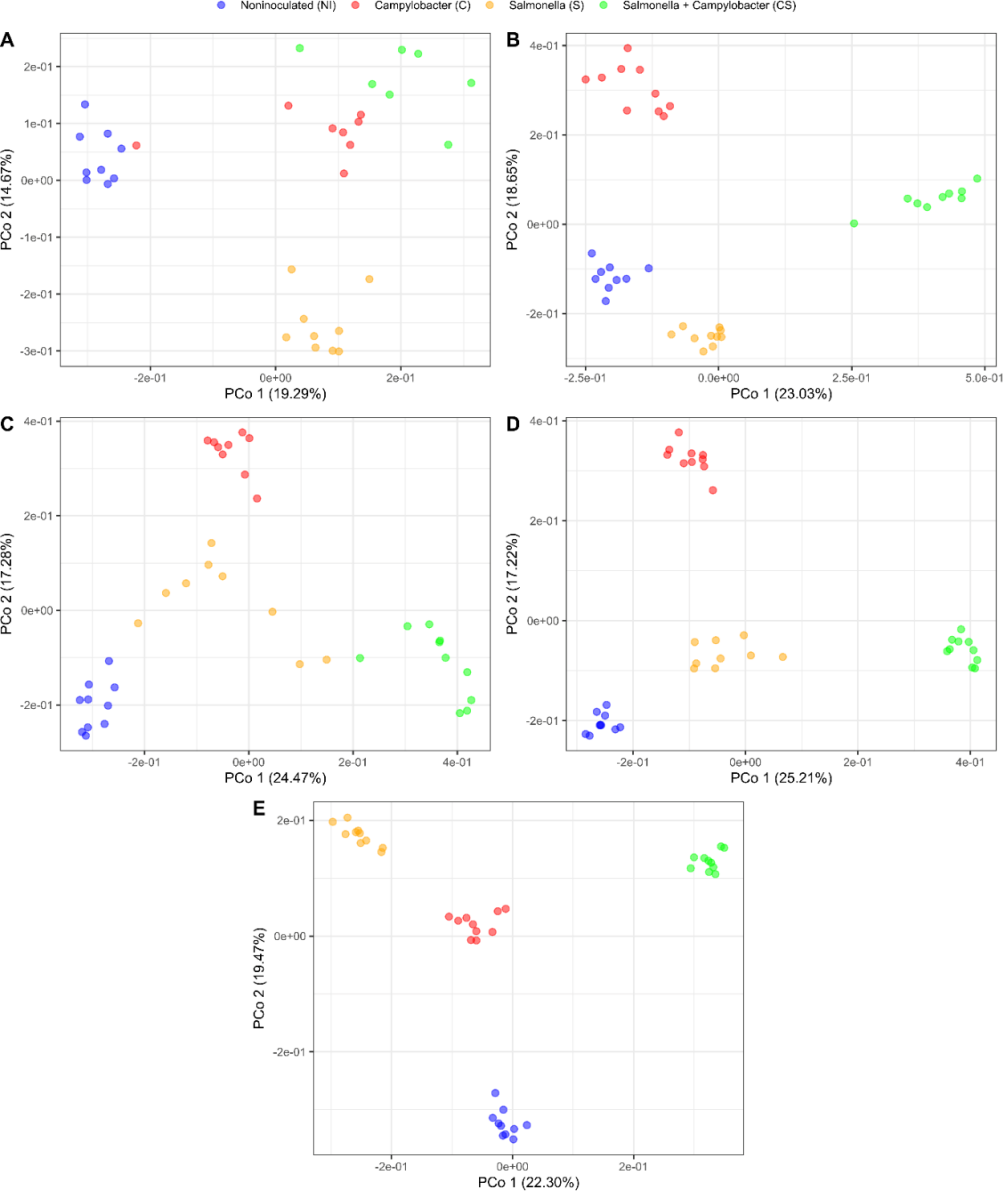
PCoA plots based on Jaccard distance of the cecal microbiota of control broilers (NI - blue), broilers challenged with *Campylobacter* (C—red), broilers challenged with *Salmonella* (S—yellow) or broilers challenged with *Campylobacter + Salmonella*(C + S—green) at (**A**) week 1, (**B**) week 2, (**C**) week 3, (**D**) week 4, and (**E**) week 5.

Analysis of α-diversity within samples was carried out using the number of observed ASVs and the Shannon index to estimate species richness and evenness. In the four groups, α-diversity increased throughout the study with the age of the animals (Table 2). Throughout the trial, α-diversity remained relatively stable in the group challenged with *Campylobacter* (C) compared to the non-infected group (NI) (Table 2). In contrast, a lower α-diversity was observed in the group exposed to *Salmonella* (S) compared to NI at week 4 (fewer ASVs) and week 5 (fewer ASVs and a lower Shannon Index) (Table 2). In the group exposed to both pathogens (C + S), notable changes in α-diversity were observed starting from the second week of the trial. Specifically, a significant decrease in the number of observed ASVs and Shannon Index was observed at week 2 in the C + S challenged group, compared to the other three groups. At week 3, animals challenged with C + S showed a decrease in α-diversity (number of observed ASVs and Shannon Index) compared to the NI and C groups, but not compared to the S group. At week 4, the number of observed ASVs was lower in the C + S group compared to NI, and the Shannon Index was significantly lower in the C + S group than in the other three groups (Table 2). At week 5, the only statistically significant difference observed in the C + S group was an increase in the Shannon Index compared to the S group. β diversity analysis was used to assess the microbial structure of the cecum from the broilers. Multi-dimensional scaling of the Jaccard distance was performed to create a visualization of differences in microbial population structures among the four groups (Fig. 4). As shown in Fig. 4, the three challenged groups clustered from each other and separately from the control group (NI). A multivariate ANOVA (PERMANOVA performed with ADONIS) revealed a significant difference in microbial populations between the four groups throughout the study (*P* < 0.001).

**TABLE 2:** Changes in α-diversity indices of the cecal microbiota following *Campylobacter* or *Salmonella* challenged alone or in combination^*a*^

Week and parameter	NI	C	S	C + S
Week 1				
Observed ASVs	146.3 ± 9.5^a^	120.3 ± 10.6^a^	139.7 ± 6.8^a^	111.0 ± 9.8^a^
Shannon index	2.55 ± 0.06^a^	2.38 ± 0.08^a^	2.59 ± 0.05^a^	2.45 ± 0.13^a^
Week 2				
Observed ASVs	192.9 ± 9.2^a^	190.6 ± 8.3^a^	206.8 ± 8.4^a^	131.2 ± 8.2^b^
Shannon index	3.10 ± 0.12^a^	3.33 ± 0.06^a^	3.35 ± 0.06^a^	2.56 ± 0.08^b^
Week 3				
Observed ASVs	224.8 ± 8.5^a,b^	249.2 ± 10.5^a^	191.2 ± 19.1^b,c^	150.1 ± 11.1^c^
Shannon index	3.64 ± 0.07^a,b^	3.73 ± 0.06^a^	3.32 ± 0.14^b,c^	3.01 ± 0.06^c^
Week 4				
Observed ASVs	251.2 ± 6.3^a^	231.2 ± 5.2^a,b^	220.0 ± 8.3^b^	208.0 ± 5.4^b^
Shannon index	3.83 ± 0.05^a^	3.79 ± 0.07^a^	3.75 ± 0.07^a^	3.47 ± 0.05^b^
Week 5				
Observed ASVs	323.6 ± 9.2^a^	314.8 ± 11.0^a^	271.5 ± 3.6^b^	292.9 ± 5.9^a,b^
Shannon index	4.11 ± 0.06^a^	3.89 ± 0.08^a^	3.22 ± 0.08^b^	3.84 ± 0.08^a^

^*a*^ Observed ASVs and Shannon indexes obtained for samples from the 4 groups were compared using a one-way ANOVA test, after verifying that distribution was normal, followed by a Bonferonni’s multiple comparison test. Different superscript letters indicate a significant difference (*P *< 0.05) between the groups. Broilers non-inoculated (NI) or challenged with *Campylobacter* (C), with *Salmonella* (S), or with *Campylobacter* + *Salmonella* (C+S) at weeks 1, 2, 3, 4, and 5 post-inoculation (data are mean ± SEM).

The LefSE analysis identified 84 bacterial genera with a significantly increased or decreased relative abundance in the challenged groups (C, S, or C + S) compared to the NI group during the 5 weeks (Table S2). Table 3 presents the bacterial genera with a relative abundance greater than 0.3% (in individual broilers), showing the same modulation (increase or decrease) for two consecutive weeks in at least one of the challenged groups. Focusing on these data helped to identify common changes in the composition of the microbiota in the three contaminated groups, compared to the control group (NI). Among them, the relative abundance of the *Klebsiella* genus from the *Enterobacteriaceae* family increased in the three challenged groups (C, S, and C + S) compared to the NI group during the first 3 weeks after the inoculation. On the contrary, several members of the *Firmicutes* phylum presented decreased abundances in the three challenged groups compared to the control group from the second week of the trial. For example, among the *Lachnospiraceae* family, a decrease in *Tyzzerella* was observed in the three challenged groups from week 2 to week 4; *Shuttleworthia* from week 3 to week 4, and *Lachnospiraceae* FE2018 group from week 3 to week 5. In the same way, *Negativillus* (Ruminococcaceae family) presented decreased abundance from week 3 to week 5.

**TABLE 3:** Bacterial genera from the cecal microbial communities of broilers challenged with *Campylobacter* (C), *Salmonella* (S), or *Campylobacter + Salmonella* (C + S) compared to control broilers (NI) at 1, 2, 3, 4, and 5 weeks post-inoculation identified by linear discrimination analysis coupled to effect size (LefSE)^*a*^

Phylum	Family	Genus	C					S					C + S				
			Week					Week					Week				
			1	2	3	4	5	1	2	3	4	5	1	2	3	4	5
*Actinobacteria*	*Bifidobacteriaceae*	*Bifidobacterium*											+	+	+		
*Campylobacterota*	*Campylobacteraceae*	*Campylobacter*		+	+	+							+	+	+	+	+
*Firmicutes*	*Erysipelatoclostridiaceae*	*Erysipelatoclostridium*	+	+	+												
	*Enterococcaceae*	*Enterococcus*												+	+	+	
	*Lactobacillaceae*	*Pediococcus*			−	−											
	*Lachnospiraceae*	*Eubacterium hallii* group									−	−					
		*Agathobacter*						−	−	−			−	−	−		
		*Anaerostipes*			+	+	+							+	+		
		*Blautia*							+	+	+	+		+	+	+	
		GCA-900066575						+	+						−	−	−
		*Lachnoclostridium*		−	−	−					−	−					
		*Lachnospiraceae* FE2018 group			−	−	−			−	−	−			−	−	−
		*Sellimonas*														−	−
		*Shuttleworthia*			−	−				−	−	−			−	−	−
		*Tyzzerella*		−	−	−				−	−	−		−	−	−	−
	*Monoglobaceae*	*Monoglobus*				−	−									−	−
	*Butyricicoccaceae*	*Butyricicoccus*						−	−	−			−	−	−	−	
	*Oscillospiraceae*	*Colidextribacter*													−	−	
		*Flavonifractor*			−	−	−										
		*Intestinimonas*				−	−										
		UCG-005				+	+				+	+					
	*Ruminococcaceae*	DTU089			−	−	−								−	−	
		*Faecalibacterium*									+	+					
		*Negativibacillus*			−	−	−			−	−	−			−	−	−
*Proteobacteria*	*Enterobacteriaceae*	*Klebsiella*	+	+	+			+	+	+			+	+	+		

^*a*^ The “+” symbol indicates higher abundance in the challenged group compared to the control group. The “−” symbol indicates lower abundance in the challenged group compared to the control group.

In addition, a similar modulation of several genera was observed in the S and C + S groups compared to the NI group. For example, an increase in *Blautia* and, on the contrary, a decrease in *Agathobacter* and *Butyricicoccus* was observed in these two groups. In the same way, *Campylobacter* and *Anaerostipes* increased, while *Monoglobus* and DTU089 decreased in the C and C + S groups compared to the NI group.

Several changes also appeared to be specifically associated with the inoculated bacteria group in comparison to the control group. The presence of *Campylobacter* alone was specifically associated with the decreased relative abundance of *Pediococcus* from week 3 to week 4. The presence of *Salmonella* alone was associated with decreased relative abundance of *Eubacterium hallii* group and increased relative abundance of *Faecalibacterium* from week 4 to week 5. The simultaneous presence of *Campylobacter* and *Salmonella* was associated with specific modulations of *Enterococcus* from week 2 to week 4 and *Colidextribacter*, *Sellimonas,* and GCA-900066575 from week 3 to week 5.

### Comparison of metabolic serum based on contamination status

After processing of the raw LC-MS data, five outlier samples were identified and removed from the data set, leaving a total of 180 samples distributed in the four groups and collected over 5 weeks to be used for the metabolomic statistical analysis (Table S3). Of the 3,900 detected metabolic signals, 250 varied significantly in at least one of the experimental groups at week 1, 661 at week 2, 347 at week 3, 371 at week 4, and 444 at week 5 (Kruskal–Wallis *P* < 0.05). Only these significantly varying signals were used for further statistical analysis.

**Fig 5:**
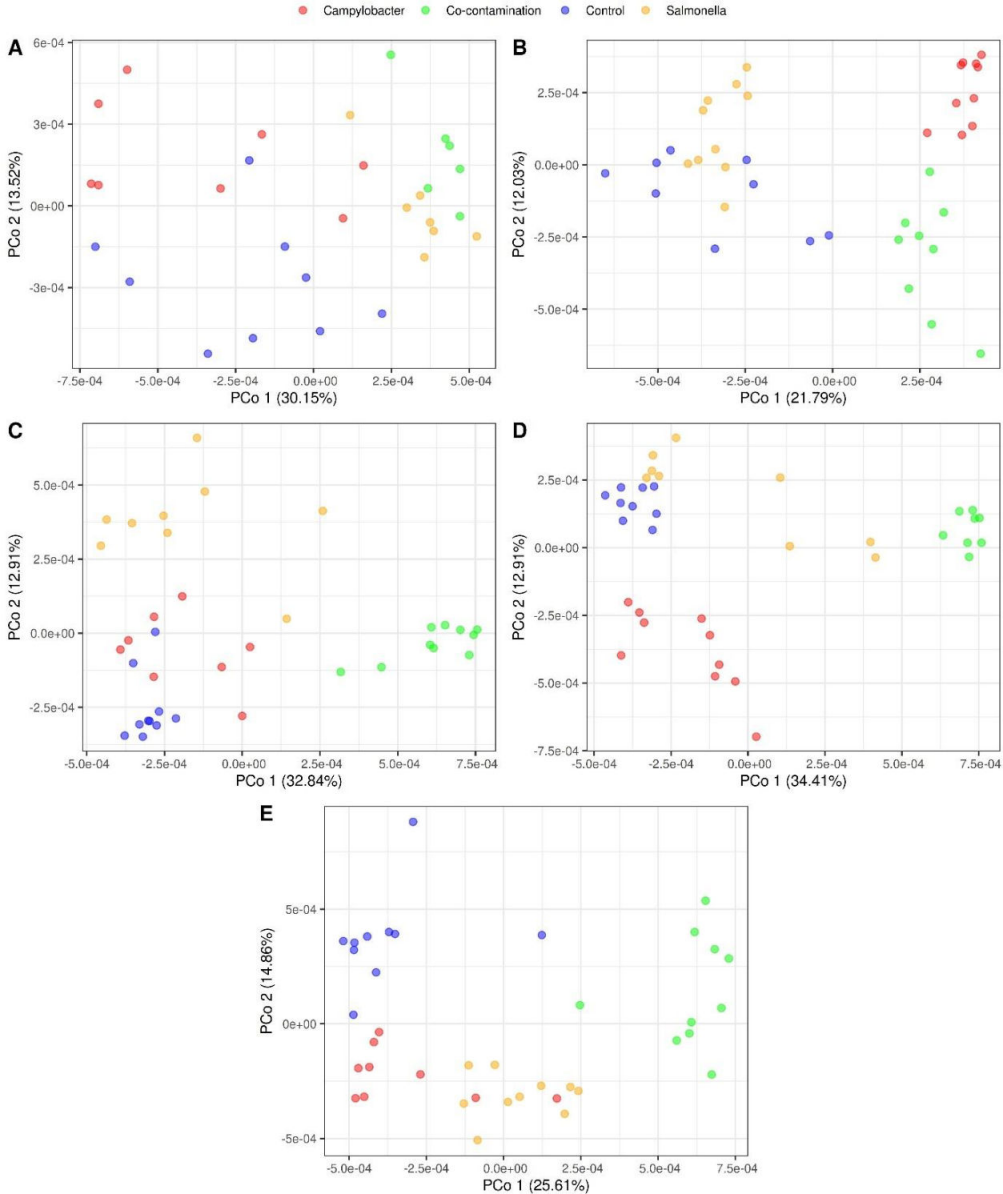
PCoA plots based on the Euclidean distance of the serum metabolome composition for samples from control broilers (NI—blue), broilers challenged with *Campylobacter* (C—red), broilers challenged with *Salmonella* (S—yellow) or broilers challenged with *Campylobacter + Salmonella* (C + S—green) at (**A**) week 1, (**B**) week 2, (**C**) week 3, (**D**) week 4, and (**E**) week 5.

Composition of the global serum metabolome was compared between the four groups (NI, C, S, and S + C) for each of the 5 weeks of the experiment. The similarity of the composition of each sample was measured using Euclidean distance and visualized using PCoA, which showed, in some cases, clear separation of the groups (Fig. 5). Statistical tests confirmed that the composition of the metabolome was at least different in one group compared to the others for each sampling week (PERMANOVA, *P* < 0.05) (Table 4). Pairwise comparisons of these four groups for each of the 5 weeks revealed that no differences in the global metabolome could be statistically measured between S and C + S, and between C and NI at week 1 (PERMANOVA, *P* > 0.05), while all the other comparisons showed significantly different global composition (PERMANOVA, *P* < 0.05) (Table 4).

**TABLE 4:** Comparison of the global composition of the serum metabolome using PERMANOVA statistical tests on Euclidean distances of broilers challenged by *Campylobacter* (C), *Salmonella* (S), or *Campylobacter* and *Salmonella* (C + S) compared to non-inoculated control broilers (NI)^*a*^

Group comparison	Week post-inoculation				
	Week 1	Week 2	Week 3	Week 4	Week 5
All groups	0.001	0.001	0.001	0.001	0.001
S/NI	0.009	0.003	0.003	0.003	0.003
C/NI	**0.195**	0.003	0.003	0.003	0.003
C + S/NI	0.012	0.003	0.003	0.006	0.003
C + S/S	**0.075**	0.003	0.003	0.006	0.003
NI/C	0.042	0.003	0.006	0.003	0.003
S/C	0.036	0.003	0.006	0.003	0.009

^*a*^ Values in bold are over the significance level (*P *= 0.05).

### PLS-DA discrimination based on contamination status

PLS-DA models were used to discriminate the samples in each of the four experimental groups based on the global metabolome. Models using the four experimental groups for the 5 weeks post-inoculation with high R2Y value were built, showing the capacity of the model to explain a large part of the variance for the training sample and a high Q2 showing that the models can achieve good discrimination of the samples in the different experimental groups (Table 5). Models generated for the discrimination of each inoculated group compared to the NI group also efficiently discriminated the samples from week 1 to week 5 (Table 5). These results demonstrate that creating PLS-DA models using global serum metabolome data enabled us to discriminate the three challenged groups from the control group with good efficiency.

**TABLE 5:** Performance values of PLS-DA models discriminating animals of the different contamination groups by their serum metabolome composition from 1 to 5 weeks post-inoculation (*P* values are in parentheses)

Group comparison	Index	Week post-inoculation				
		Week 1	Week 2	Week 3	Week 4	Week 5
All groups	R2Y (pR2Y)	0.832 (0.002)	0.873 (0.002)	0.916 (0.002)	0.968 (0.002)	0.973 (0.002)
	Q2 (pQ2)	0.629 (0.002)	0.718 (0.002)	0.740 (0.002)	0.852 (0.002)	0.765 (0.002)
S/NI	R2Y (pR2Y)	0.917 (0.026)	0.815 (0.016)	0.830 (0.002)	0.987 (0.002)	0.978 (0.002)
	Q2 (pQ2)	0.804 (0.002)	0.660 (0.002)	0.747 (0.002)	0.877 (0.002)	0.851 (0.002)
C/NI	R2Y (pR2Y)	0.906 (0.024)	0.991 (0.006)	0.990 (0.004)	0.993 (0.006)	0.990 (0.036)
	Q2 (pQ2)	0.749 (0.002)	0.933 (0.002)	0.857 (0.002)	0.837 (0.002)	0.865 (0.002)
C + S/NI	R2Y (pR2Y)	0.997 (0.002)	0.809 (0.012)	0.999 (0.002)	0.992 (0.002)	0.986 (0.002)
	Q2 (pQ2)	0.954 (0.002)	0.727 (0.002)	0.950 (0.002)	0.901 (0.002)	0.877 (0.002)

### Identification of determinant variables for discrimination of samples using bootstrapped sPLS-DA

The most important metabolic signals for the classification of the samples in the different models were identified using bootstrapped sPLS-DA models and retained as determinant variables if they were selected more than 300 times for the 500 iterations of the models. The results showed that a high number of determinant variables in the different classification models was observed for the three groups 1 week post-inoculation. Of the 250 variables used to build the models, 143 were identified as determinant variables for discrimination of the S group from the NI group, 91 for discrimination of the C group from the NI group, and 85 for discrimination of the C + S group from the NI groups (Table 6). The number of determinant variables was the highest at week 2, both for S and C + S comparisons (284 and 201 out of 661, respectively), while sharply dropping (only one variable) for discrimination of the C group from the NI group, and remaining low until the end of the experiment. The number of determinant variables for the discrimination of the S group from the NI group stayed the highest until week 4. For the C + S group, the number of variables dropped to 1 at week 3 and remained low until week 5. These results showed a different evolution in the impact of the different pathogens on the metabolic signal and that *Salmonella* inoculation had an impact on a larger number of metabolic signals than the other groups, at least for the first 4 weeks of the experiment (Table 6).

**TABLE 6:** Number of total variables kept for the production of sPLS-DA models for each week post-inoculation and the number of selected variables by these models for the discriminant analysis of the different groups of contaminated and control animals

Variables	Week 1	Week 2	Week 3	Week 4	Week 5
Total variables used for sPLS-DA	250	661	347	471	444
S/NI	143	284	81	17	1
C/NI	91	1	10	1	3
C + S/NI	85	201	1	1	4

The results also showed common important signals between the models for different inoculated groups, which were mostly found 1 week post-inoculation (Table S4).

At week 1, among the determinant signals to discriminate the C + S group from the NI group, 5 were common with those discriminating the C group from the NI group, and 11 with those discriminating the S group from the NI group. At week 2, a single determinant signal was common for the discrimination of both the S and C + S groups, while another was determinant for the discrimination of both the S and C groups. No common signals were detected for the last 3 weeks, and no common signals were identified over multiple weeks.

Moreover, the evolution of the determinant signals during the 5 weeks was also evaluated for each inoculated group by identifying metabolic signals that were selected over multiple weeks, showing marked variation of the determinant signals over time, with a limited number selected for two consecutive weeks (Table S5).

### Association between metabolic composition and cecal microbiota composition

**Fig 6:**
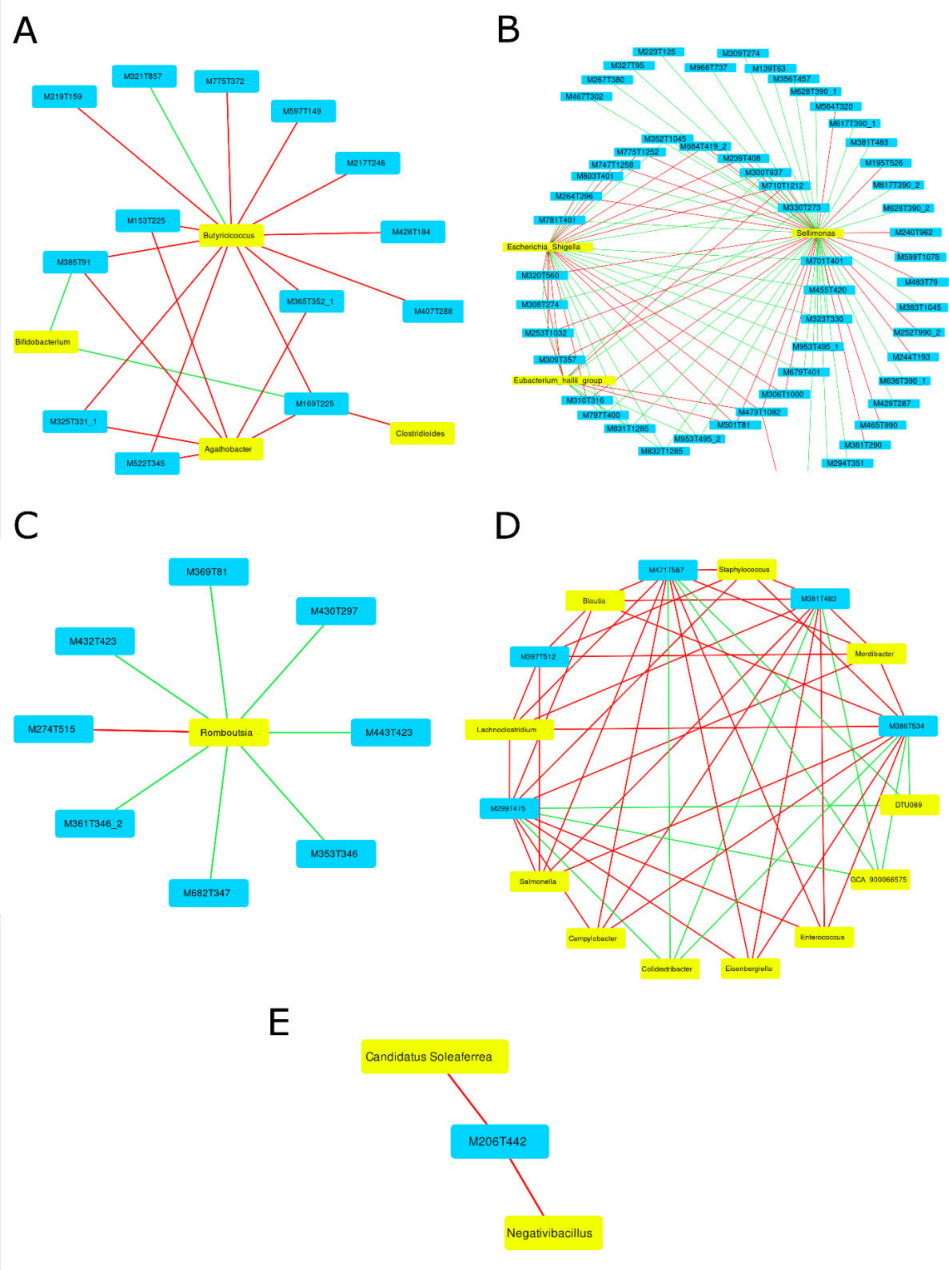
Network graph showing association between taxa (yellow) and metabolic signals (blue) with a correlation value higher than 0.45 at (**A**) week 1, (**B**) week 2, (**C**) week 3, (**D**) week 4, and (**E**) week 5 post-inoculation. A red line indicates a negative association, while a green one indicates a positive association.

Using sPLS regression models for each week, we found that variations in some taxa of the fecal microbiota can be associated with variations in the signals in the serum metabolome. In multiple cases, one taxon could be highly associated with variations of multiple metabolic signals. For example, at week 1, the *Butyricicoccus* taxon was highly associated both negatively and positively with 13 signals (Fig. 6). Inversely, multiple taxa could also be highly associated with a single metabolic signal. For example, at week 1, the metabolic signal M385T91 was associated negatively with *Butyricicoccus* and *Agathobacter* and positively associated with *Bifidobacterium*. However, these associations seem to be highly variable over the weeks, as shown in Fig. 6. Furthermore, in some cases, the presence of *Salmonella* or *Campylobacter* could be directly associated with metabolic signal variations (Table S6). Their inoculation was directly highly associated, in a negative way, with four metabolic signals each. However, these high associations were only measured 4 weeks after inoculation (Table S6). Three of these metabolic signals were common for the two pathogens.

## DISCUSSION

Inoculation of young chicks with *Campylobacter* alone resulted in high levels (6–8 log CFU/g) of colonization maintained throughout the duration of the trial. Conversely, inoculation with *Salmonella* caused an initial high level of colonization, followed by a substantial decline (more than 3 log CFU/g) at the end of the trial. Similar results were previously observed during *in vivo* trials with these two bacteria (26, 34–36). To our knowledge, this study is the first to demonstrate a reciprocal positive effect of *C. jejuni* and *S*. Typhimurium on broiler colonization during an *in vivo* experimental trial. This study revealed that *Campylobacter* levels in broiler ceca were higher when *Salmonella* was present, and the presence of *Campylobacter* prevented the reduction of *Salmonella* colonization in the broiler cecum. Similarly, a recent study demonstrated *in vitro* that *S*. Typhimurium positively affected the survival of *Campylobacter jejuni* under aerobic conditions, suggesting potential interaction between these pathogens (6). These findings underline the importance of considering both pathogens in future investigations as recommended in a European Food Safety Authority opinion on *Salmonella* control in poultry (5).

In addition to the difference in colonization, simultaneous inoculation with *C. jejuni* and *S*. Typhimurium led to a reduction in body weight during the first 4 weeks. In contrast, *Campylobacter* alone did not affect body weight, consistent with other studies at the same dose (26, 37). Similarly, except for 1 week after the challenge, *Salmonella* alone did not affect weight, as previously observed (12, 18, 38, 39).

Inoculation of the chicks with *C. jejuni* or *S. Typhimurium* or both bacteria was associated with changes in the cecal microbiota compared to the non-inoculated group. As shown in other studies, *Campylobacter* led to changes in β-diversity without affecting α-diversity throughout the study (9, 10, 40, 41). Likewise, the presence of *S*. Typhimurium alone induced a change in β-diversity from the first week post-inoculation consistent with the literature (7, 8, 11). Interestingly, a decrease in α-diversity was observed from week 4 onwards, coinciding with a reduction in *Salmonella* load. However, a previous study did not observe changes in α and β-diversity after 2–4 days of *S*. Typhimurium challenge (18). However, the *Salmonella* serotype, the chicken breed, and the time post-infection at which the microbiota was observed could be responsible for these discrepancies (7, 11, 38, 42).

The C + S group presented the most significant microbiota modifications, with changes in both β-diversity and a significant reduction in α-diversity, from the first to the fourth week post-inoculation.

Both *C. jejuni* and *S*. Typhimurium are known, individually, to modify the abundance of multiple genera in broilers (7–12, 14, 26). In the C + S group, we observed similar changes of genera common to both C and S, as well as changes specific to C or S. Specifically, there was an increased abundance of *Klebsiella* and a decreased abundance of *Tyzzerella* and *Lachnospiraceae FE2018* group as observed in C and S. Opposite results, regarding the variation of abundance of several genera, were observed, such as for *Butyricicoccus* and *Faecalibacterium*. For example, a decreased abundance of *Butyricicoccus* was observed from week 1 to week 3 in the S group and from week 1 to week 5 in C + S, where *Salmonella* loads were high throughout the trial. On the contrary, a higher abundance of *Faecalibacterium* was observed from week 4 to week 5, when *Salmonella* loads in this group were lower. These differences might be linked to varying *Salmonella* loads in S and C + S, as observed by Khan et al*.* (7).

Additionally, bacterial genera showed specific modulation over two consecutive weeks in the co-contaminated group. For example, the genera *Sellimonas*, *Colidextribacter,* and GCA-900066575 were reduced in co-contaminated broilers compared to non-inoculated controls in the last 3 weeks of the study, suggesting that they may be involved in maintaining *Salmonella* and/or *Campylobacter* in the co-contaminated group.

This study also suggests that inoculation with *S*. Typhimurium and/or *C. jejuni* led to significant changes in the global serum metabolome from the first week after inoculation until the end of the study at 5 weeks. PLS-DA models successfully discriminated contaminated and non-contaminated animals based on metabolic signals, some of which were linked to microbiota taxa variations.

The significant metabolome variations observed in the S group were consistent with a previous study, where *Salmonella* Enteritidis affected the cecum metabolome from 3 days post-inoculation (dpi) to 21 dpi (25). These changes were attributed to a metabolic switch, with a shift from the tricarboxylic acid cycle to aerobic glycolysis due to innate immune system activation (25).

Interestingly, *Campylobacter* presence was correlated with delayed changes in the metabolome, observed 2 weeks after inoculation. Another study showed that *Campylobacter* inoculation of chicks at 7 days of age caused changes in the serum metabolome 7 days after inoculation, with increased level of molecules associated with the innate immune system (43). The discrepancy between the two studies could be explained by the maturity of the immune system or the microbiota of the animals, bearing in mind that at the beginning of life, the metabolic response to *Campylobacter* is reduced (44, 45).

Despite a higher abundance of *Campylobacter* in the cecum compared to *Salmonella*, the associated changes in the global metabolome were less pronounced or occurred later. Even without significant global metabolome changes, *Campylobacter*-inoculated animals were distinguished using PLS-DA models, suggesting more subtle metabolic effects than those caused by *Salmonella*. In both cases, the number of determinant variables for sample discrimination samples was high in the first 2 weeks after inoculation; then these determinant signals varied over time. These results may reflect a strong impact of inoculation at the beginning of the experiment, leading to an effect on a large number of metabolites, which transitions to more homeostatic asymptomatic carriage later in the animals’ lifespan (46). The rapid decrease in the number of determinant signals in the C group could be attributed to *Salmonella*’s stronger interaction with the chicken intestinal mucosa, which results in more prolonged changes in the metabolome (47). These results further suggest that the impact of *Salmonella* inoculation could have a stronger effect on the chicken organism, possibly by activation of inflammatory mechanisms not mobilized in the case of *Campylobacter*. Only one signal was found to be common between the S and C groups over the 5 weeks (at week 2) of the experiment, highlighting the distinct effects of these pathogens on the animals’ metabolome. These differences might be due to differential activation of host homeostasis mechanisms or microbiota modifications following inoculation. Significant differences in the global metabolome were also observed between the C + S and NI groups. The serum metabolome of the C + S group differed significantly from the metabolome from the C group, but not from that of the S group, suggesting that *Salmonella* largely influenced the early effects measured in the co-inoculated animals. This is consistent with the rapid metabolic response typically seen following *Salmonella* infection (25).

The most common determinant signals were found between the C + S group and one of the other contaminated groups (S or C). While a major part of the effect of co-infection on the metabolome was unique to this group, some of the effects due to the presence of *Salmonella* and *Campylobacter* alone could also be detected, especially in the first 2 weeks post-inoculation. These results suggest that the early impact of co-inoculation resembled the combined effects of both pathogens. Over time, the impact diverged into a single effect due to synergy between the two pathogens. These findings are similar to those observed in the microbiota.

Finally, this study showed that variations in specific cecal microbiota taxa were associated with changes in metabolic signals considered to be important for discriminant analysis models. These results are supported by the literature, which indicates that gut microbiota changes can be associated with variations of the metabolome in the gut but also at the systemic level (20–24, 29). In this study, the associations detected were not conserved over multiple weeks, possibly reflecting the evolution of both the microbiota and the metabolome over time, as discussed previously. Interestingly, at week 1 post-inoculation, *Bifidobacterium*, *Butyricicoccus,* and *Agathobacter* were associated with metabolic signals. These genera are known for producing SCFAs, which were described as protective against pathogen colonization (48–51). *Salmonella* and *Campylobacter* were strongly associated with variations in specific metabolic signals only at week 4, suggesting a non-linear association with most of the metabolic signal changes. Hence, it seems that in response to inoculation, most of the changes in the metabolome are not the result of the direct production of metabolites by the pathogens, but of complex interactions linked to changes in the global microbiota or in the metabolism of the animal. These results highlight the complex dynamics of host-microbiota-pathogen interactions and confirm the need to design and implement further studies to identify the molecules that are represented by these metabolic signals, to better understand the nature of these associations between microbiota and metabolome in the context of inoculation by these pathogens.

### Conclusion

This study demonstrates the reciprocal impact of *Campylobacter* and *Salmonella* on broiler cecum colonization and emphasizes the need for adapted control measures in the poultry industry. The research also provides a deeper understanding of the individual and combined effects of these pathogens on the cecal microbiota, revealing unique variations associated with co-inoculation. Furthermore, the study highlights sustained and clinically silent changes in the serum metabolome of broilers following *Salmonella*, *Campylobacter,* and co-inoculation, persisting for up to 5 weeks. The ability to distinguish contaminated from non-contaminated animals based on metabolic signatures underscores the diagnostic potential of serum metabolomics. Finally, the study underscores the complex interplay between pathogen presence, microbial composition, and metabolic changes. These findings not only address critical gaps in the understanding of *Campylobacter* and *Salmonella* interactions in poultry but also pave the way for future research into the molecular mechanisms underlying these interactions.

## Data Availability

The data from this study have been deposited in the NCBI repository under BioProject accession number PRJNA1246375 and Sequence Read Archive (SRA) accession numbers SRX29717819–SRX29718008.

## SUPPLEMENTAL MATERIAL

Fig. S1 spectrum.01102-25-s0001.tif Relative abundance of the 6 main genera in cecal microbiota.

Supplemental tables spectrum.01102-25-s0002.xlsx Tables S1 to S6.

ASM does not own the copyrights to Supplemental Material that may be linked to, or accessed through, an article. The authors have granted ASM a non-exclusive, world-wide license to publish the Supplemental Material files. Please contact the corresponding author directly for reuse.
