# Supplementary figures and images for: Co-inoculation of broilers by Campylobacter and Salmonella: effect on colonization, cecal microbiota, and serum metabolome
Source: Microbiol Spectr. 2026 Feb 6;14(3):e01102-25. doi: 10.1128/spectrum.01102-25 (PMC12955424; doi:10.1128/spectrum.01102-25)

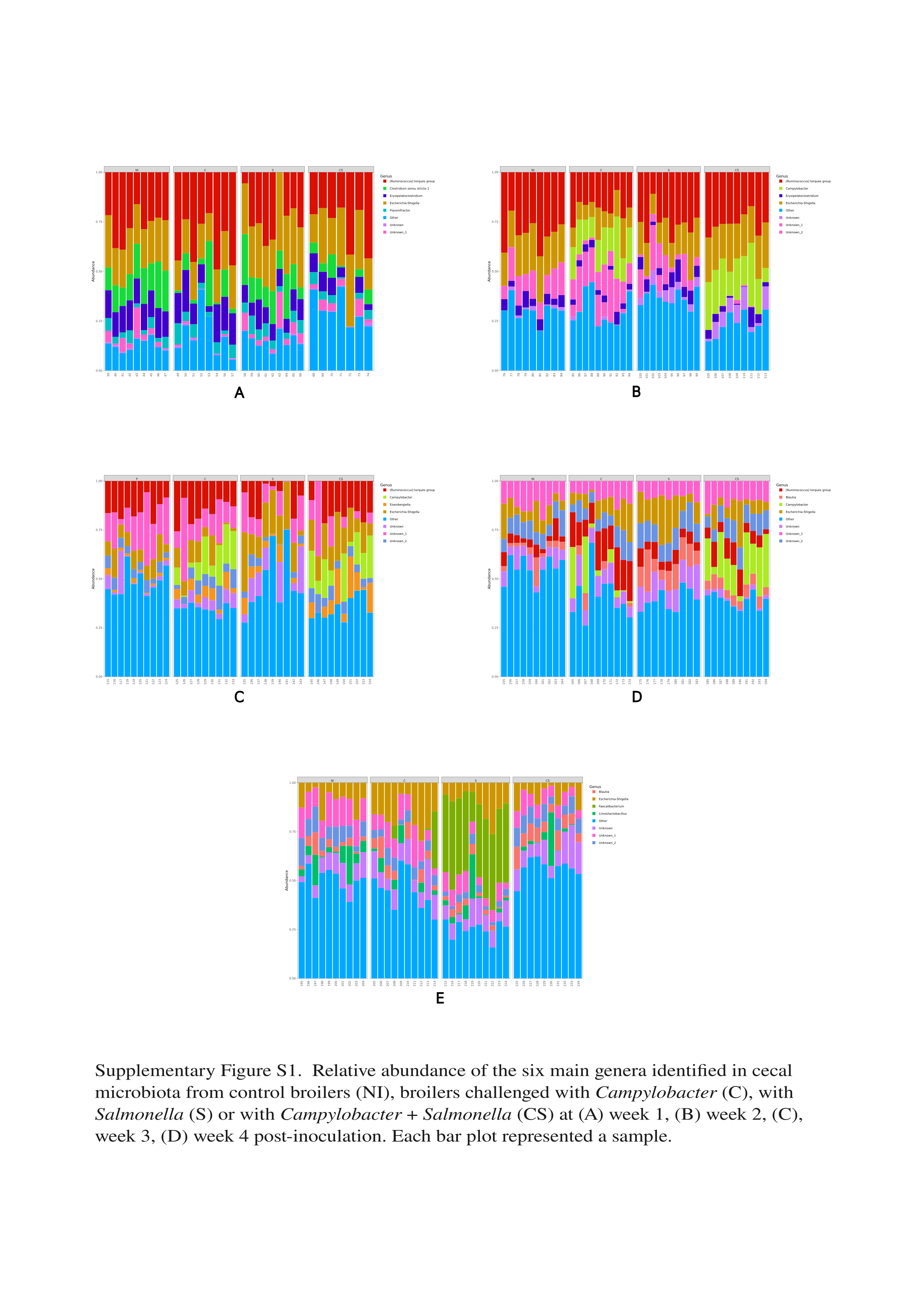

Supplement: Fig. S1 — Relative abundance of the 6 main genera in cecal microbiota. [file spectrum.01102-25-s0001.tif]
